# Novel immune-related genes in the tumor microenvironment with prognostic value in breast cancer

**DOI:** 10.1186/s12885-021-07837-1

**Published:** 2021-02-06

**Authors:** Wen Tan, Maomao Liu, Liangshan Wang, Yang Guo, Changsheng Wei, Shuqi Zhang, Chengyu Luo, Nan Liu

**Affiliations:** 1 Center for Cardiac Intensive Care, Beijing Anzhen Hospital, Capital Medical University, Beijing, China; 2 Department of General Surgery, Beijing Anzhen Hospital, Capital Medical University, Beijing, China

**Keywords:** Breast cancer, Tumor microenvironment, Immune-related gene, Prognostic signature, Immunotherapy

## Abstract

**Background:**

Breast cancer is one of the most frequently diagnosed cancers among women worldwide. Alterations in the tumor microenvironment (TME) have been increasingly recognized as key in the development and progression of breast cancer in recent years. To deeply comprehend the gene expression profiling of the TME and identify immunological targets, as well as determine the relationship between gene expression and different prognoses is highly critical.

**Methods:**

The stromal/immune scores of breast cancer patients from The Cancer Genome Atlas (TCGA) were employed to comprehensively evaluate the TME. Then, TME characteristics were assessed, overlapping genes of the top 3 Gene Ontology (GO) terms and upregulated differentially expressed genes (DEGs) were analyzed. Finally, through combined analyses of overall survival, time-dependent receiver operating characteristic (ROC), and protein-protein interaction (PPI) network, novel immune related genes with good prognosis were screened and validated in both TCGA and GEO database.

**Results:**

Although the TME did not correlate with the stages of breast cancer, it was closely associated with the subtypes of breast cancer and gene mutations (CDH1, TP53 and PTEN), and had immunological characteristics. Based on GO functional enrichment analysis, the upregulated genes from the high vs low immune score groups were mainly involved in T cell activation, the external side of the plasma membrane, and receptor ligand activity. The top GO terms of the upregulated DEGs from the high vs low immune score groups exhibited better prognosis in breast cancer; 15 of them were related to good prognosis in breast cancer, especially CD226 and KLRC4-KLRK1.

**Conclusions:**

High CD226 and KLRC4-KLRK1 expression levels were identified and validated to correlate with better overall survival in specific stages or subtypes of breast cancer. CD226, KLRC4-KLRK1 and other new targets seem to be promising avenues for promoting antitumor targeted immunotherapy in breast cancer.

**Supplementary Information:**

The online version contains supplementary material available at 10.1186/s12885-021-07837-1.

## Background

Breast cancer is one of the most frequently diagnosed cancers among women worldwide [1]. With improvements in early detection and treatment, the number of deaths from breast cancer has continuously declined since 1990 [2]. However, in the United States, an estimated 268,600 women will still be diagnosed with breast cancer, and 41,760 women will die of breast cancer in 2019 [3].

Multidisciplinary approaches, including chemotherapy, endocrine therapy, molecular targeted therapy, radiotherapy, and surgery, are commonly used to improve breast cancer patient survival. However, not all patients benefit from combined treatment, and as many as 40% of women with breast cancer will still be resistant to currently available targeted therapy approaches [4]. At present, numerous issues remain unresolved, and there is a lack of adequate evidence to fully clarify the mechanism of breast tumorigenesis. In recent years, obvious alterations in the tumor microenvironment (TME) have been increasingly recognized as key in the development and progression of breast cancer.

The TME consists of tumor cells, stromal cells (such as fibroblasts, endothelial cells, pericytes, myoepithelial cells, and adipocytes), immune cells (such as macrophages, dendritic cells, natural killer (NK) cells, T lymphocytes, and B lymphocytes), extracellular matrix (ECM), signaling molecules and cytokines [5, 6]. Stromal cells and immune cells are two major types of nontumor components in the TME, so the stromal or immune components are proposed to be valuable for diagnostic and prognostic assessments of the TME. It was demonstrated that epigenetic alterations generating aberrant gene expression in the cells of the TME are predictive of clinical outcomes [7]. In addition, there is increasing interest in the breast cancer microenvironment as a prognostic factor as well as a potential therapeutic target; thus, a wider assessment of immune responses within the TME by gene expression profiling might effectively predict clinical outcomes [8]. A deeper comprehension of the genetic profile of the breast cancer TME is urgently needed.

In this study, comprehensive bioinformatics analyses were performed to better understand the immune-related genetic profile and determine the relationship between gene expression in the microenvironment of breast cancer and prognosis. Our study will provide applicable information on novel immunological targets from the TME, complement other treatment options for breast cancer and improve the overall survival (OS) of patients.

## Methods

### Data sources and preprocessing

RNA sequencing (RNA-seq) data in fragments per kilobase of transcript per million mapped reads (FPKM), single-nucleotide polymorphism data and clinical follow-up information such as age, molecular subtype, outcome and survival time of breast cancer were downloaded from The Cancer Genome Atlas (TCGA) database. A total of 1097 cases were obtained from the TCGA dataset. Then, the RNA-seq data were converted into transcripts per kilobase million (TPM) expression profiles. The immune and stromal scores of the samples were calculated by the Estimation of STromal and Immune cells in MAlignant Tumor tissues using Expression data (ESTIMATE) algorithm via the ESTIMATE R software package. Next, two series of gene expression profiles from the Gene Expression Omnibus (GEO) database (GSE20685 and GSE42568) with clinical data from 431 breast cancer patients were used as the validation datasets. The GEO data were downloaded and normalized by the LIMMA R software package.

### Comprehensive assessment of the TME characteristics in breast cancer

Based on the mean stromal or immune scores as the cutoff value, the patients were divided into two groups: the high stromal/immune score group and the low stromal/immune score group. Then, we analyzed the relationships between different stages/subtypes of breast cancer and stromal/immune scores. Kaplan-Meier (K-M) analysis was performed to determine the differences in OS between the high- and low-score groups of the four subtypes of breast cancer. In addition, we detected the correlations between stromal/immune scores and conventional gene mutations.

### Identification of differentially expressed genes (DEGs)

Following the high and low score grouping, we screened for DEGs to obtain those with a false discovery rate (FDR) < 0.05 and a log2 fold change (FC) > 1. Heatmaps and cluster maps of the DEGs were generated using the Pheatmap package, and volcano plots were designed by the ggplot2 package in R software. Then, the upregulated genes with high vs low scores were further analyzed with Gene Ontology (GO) and Kyoto Encyclopedia of Genes and Genomes (KEGG) pathway analysis. GO categories including biological process (BP), molecular function (MF), or cellular component (CC) were analyzed using the EnrichGO function in the clusterProfiler R package and KEGG analysis was performed by the EnrichKEGG function in the clusterProfiler R package, with the parameters pvalue-Cutoff = 0.05 and qvalue-Cutoff = 0.05. A Venn diagram was used to screen the overlapping genes between the upregulated DEGs from high vs low scores and the top 3 GO terms by the online tool VENNY 2.1 (https://bioinfogp.cnb.csic.es/tools/venny/index.html).

### Construction of the protein-protein interaction (PPI) network

The PPI network was established in the Search Tool for the Retrieval of Interacting Genes/Proteins (STRING) database and reanalyzed by Cytoscape software. The medium confidence was set as 0.4. Only individual networks with 10 or more nodes were included for further analysis. The connectivity degree of each node of the network was calculated. The logFC value of the gene expression was used to reflect the color of each node, and the size of the node represented the number of proteins interacting with the designated protein. Molecular COmplex Detection (MCODE) was then used to locate the central gene cluster.

### Screening immune-related genes with prognostic value

To identify genes with prognostic value in the TME, we performed univariate survival analysis and used K-M analysis to analyze the relationship between the expression of these genes and the OS of breast cancer patients. Then, a time-dependent receiver operating characteristic (ROC) curve was generated to assess the predictive accuracy.

### Statistical analysis

All data in the present study were performed in R software (version 3.6.1; https://www.r-project.org/). The stromal or immune scores were calculated by ESTIMATE package. Survival curve was constructed by K-M analysis and compared by log-rank test, which was done to explore the survival differences in subtypes or stages of breast cancer and to identify the prognosis of selected genes using the GraphPad Prism 6.0 software (GraphPad Software Inc., La Jolla, CA, USA). Heatmaps, volcano plots, GO enrichment, KEGG pathway and Time-dependent ROC curve were plotted by R software. Time-dependent ROC curve as done with the TimeROC package. The confidence index of AUC was calculated by combined with Survival and TimeROC package. All statistical tests were two-sided and *P*-value< 0.05 was considered statistically significant.

## Results

### Immune scores and stromal scores are closely related to breast cancer subtypes but not associated with breast cancer stages

**Fig. 1:**
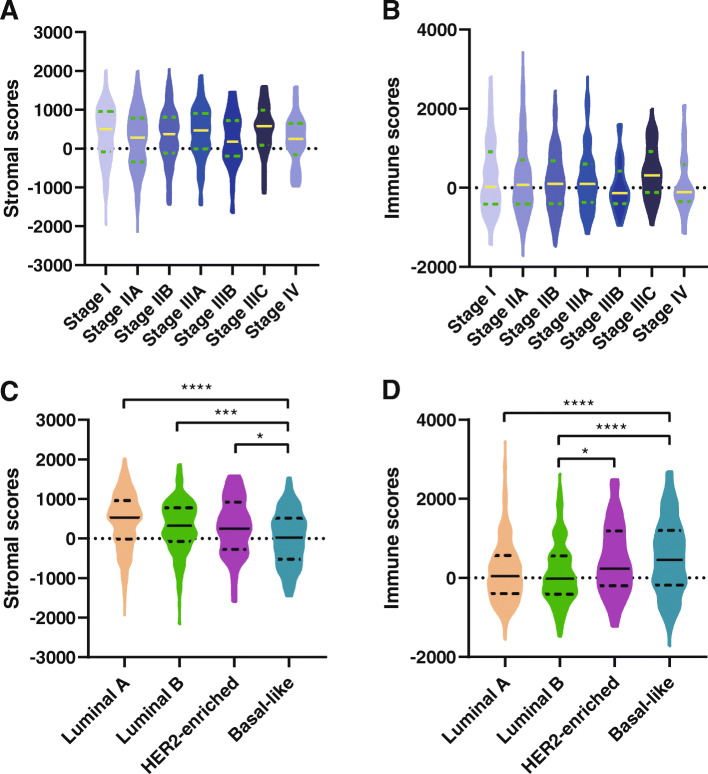
Stromal scores and immune scores were closely correlated with breast cancer. (**A**) Violin plot of stromal scores in different stages of breast cancer; (**B**) violin plot of immune scores in different stages of breast cancer; (**C**) violin plot of stromal scores in four subtypes of breast cancer; (**D**) violin plot of immune scores in four subtypes of breast cancer. *p ≤ 0.05; **p ≤ 0.01; ***p ≤ 0.001; and ****p ≤ 0.0001

In our study, the stromal scores ranged from − 2164.14 to 2050.55, and the immune scores were distributed between − 1724.88 and 3459.35. First, we assessed the relationships between stromal scores or immune scores and different stages of breast cancer. However, neither stromal scores nor immune scores showed significant differences in different stages of breast cancer (Fig. 1a, b). Then, we explored whether stromal scores or immune scores were correlated with breast cancer subtypes. The average stromal scores of the basal-like subtype were the lowest of all four subtypes (Fig. 1c); however, the average immune scores of the basal-like subtype ranked highest (Fig. 1d). In addition, the immune scores of the HER2-enriched subtype were higher than those of the luminal B subtype (Fig. 1d). Finally, we divided the scores into high and low groups and evaluated the survival probabilities of distinct subtypes in both groups of stromal scores and immune scores. Unfortunately, the high- and low-score groups did not show any survival differences in the four breast cancer subtypes (Fig. S1).

### Breast cancer gene mutations were correlated with stromal scores or immune scores

**Fig. 2:**
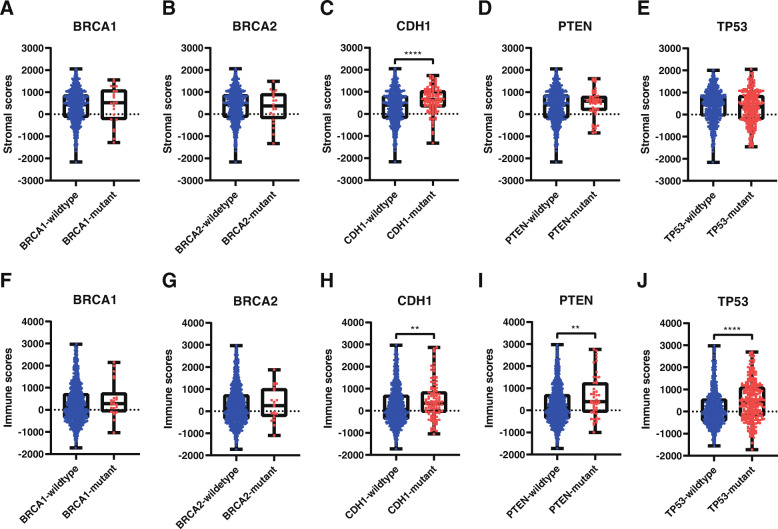
Gene mutations were associated with stromal scores and immune scores. (**A**) Distribution of stromal scores for BRCA1 mutant and BRCA1 wildtype; (**B**) distribution of stromal scores for BRCA2 mutant and BRCA2 wildtype; (**C**) distribution of stromal scores for CDH1 mutant and CDH1 wildtype; (**D**) distribution of stromal scores for PTEN mutant and PTEN wildtype; (**E**) distribution of stromal scores for TP53 mutant and TP53 wildtype; (**F**) distribution of immune scores for BRCA1 mutant and BRCA1 wildtype; (**G**) distribution of immune scores for BRCA2 mutant and BRCA2 wildtype; (**H**) distribution of immune scores for CDH1 mutant and CDH1 wildtype; (**I**) distribution of immune scores for PTEN mutant and PTEN wildtype; (**J**) distribution of immune scores for TP53 mutant and TP53 wildtype. *p ≤ 0.05; **p ≤ 0.01; ***p ≤ 0.001; and ****p ≤ 0.0001

Some gene mutations are implicated in cancer susceptibility. Thus, we downloaded single-nucleotide polymorphism data on several conventional mutant genes in the clinic, such as BRCA1, BRCA2, CHEK2, PALB2, BRIP1, TP53, PTEN, STK11, CDH1, ATM, BARD1, MLH1, MRE11A, MSH2, MSH6, NBN, MUTYH, PMS1, PMS2, RAD50, and RAD51C. Then, the patients were divided into mutant and wild-type groups, and the distributions of stromal scores and immune scores were plotted based on the status of the mutant genes in breast cancer. When compared with the wild-type group, either the BRCA1 or BRCA2 mutant group exhibited no significantly difference in the stromal scores and immune scores (Fig. 2a, b, f, g). However, CDH1 mutant cases had higher stromal scores and immune scores (Fig. 2c, h). Although stromal scores of the PTEN/TP53 mutant and wild-type groups were not statistically significant (Fig. 2d, e), PTEN/TP53 mutant cases exhibited higher immune scores than wild-type cases (Fig. 2i, j). Other mutant genes, such as CHEK2, PALB2, BRIP1, STK11, ATM, BARD1, MLH1, MRE11A, MSH2, MSH6, NBN, MUTYH, PMS1, PMS2, RAD50, and RAD51C, had no significant differences in stromal scores or immune scores between the mutant and wild-type groups (data not shown). Thus, CDH1, PTEN and TP53 mutations were closely associated with stromal/immune scores in breast cancer.

### Differential gene expression profiles with stromal scores and immune scores in breast cancer

**Fig. 3:**
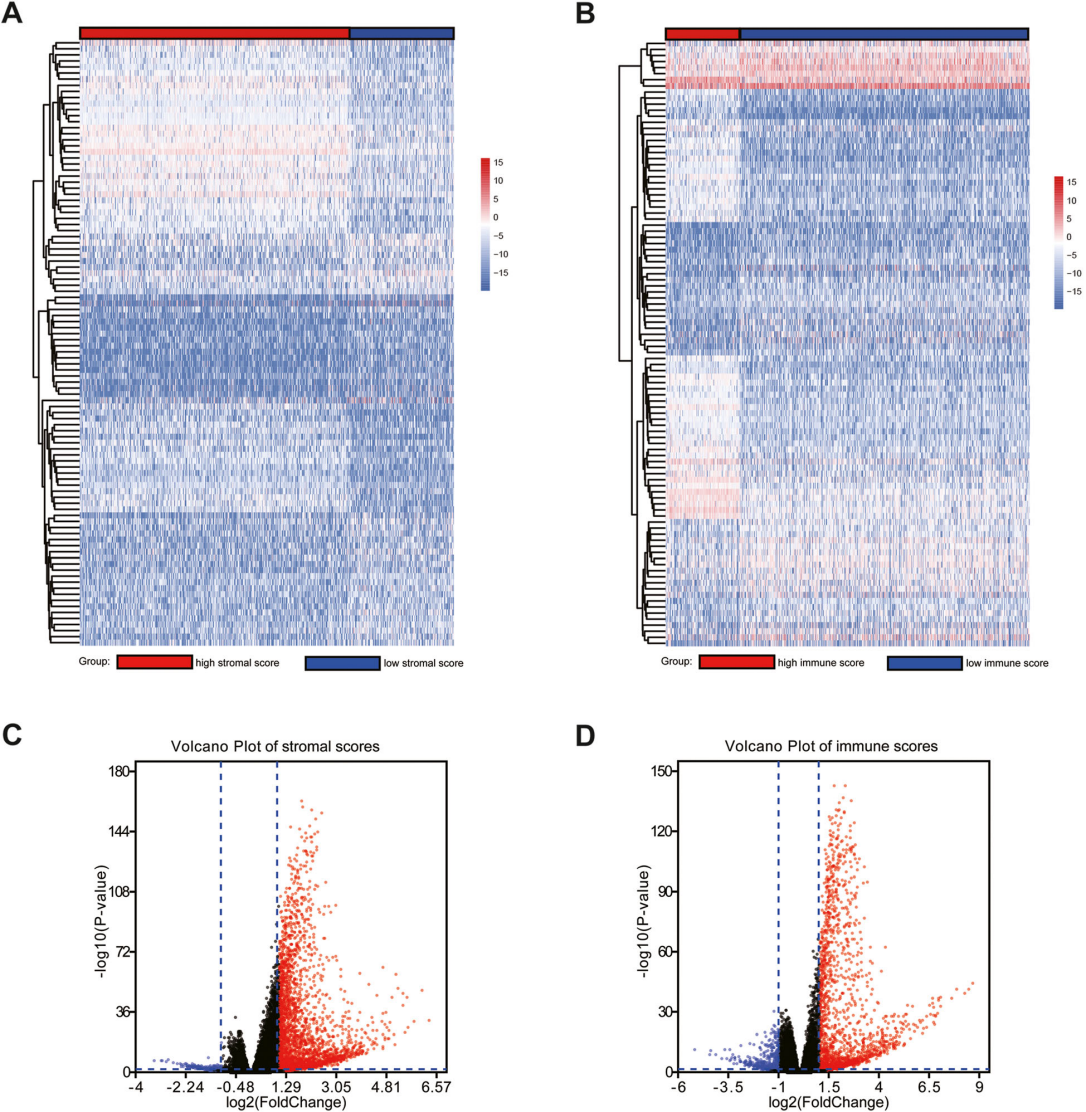
Differential gene expression profiles in breast cancer and immune score and stromal score grouping. (**A**) Heatmap of the top 100 upregulated genes in the high vs low stromal score groups; (**B**) heatmap of the top 100 upregulated genes in the high vs low immune score groups; (**C**) volcano plot of differential genes in the high vs low stromal score groups; (**D**) volcano plot of differential genes in the high vs low immune score groups

To determine the correlation between comprehensive gene expression profiles and stromal/immune scores, we analyzed the 1097 cases acquired from the TCGA database. The heatmap shows the top 100 distinct gene expression profiles of cases belonging to the high vs. low score groups (Fig. 3a, b). Based on the stromal score grouping, 2832 genes were upregulated and 253 genes were downregulated in the high vs. low stromal score groups. Similarly, 1930 genes were upregulated and 491 genes were downregulated in the high vs. low immune score groups (*p* < 0.05, log2 FC > 1). Figure 3c and d show the volcano plot of differentially expressed genes (DEGs) in high vs. low stromal/immune score groups.

**Fig. 4:**
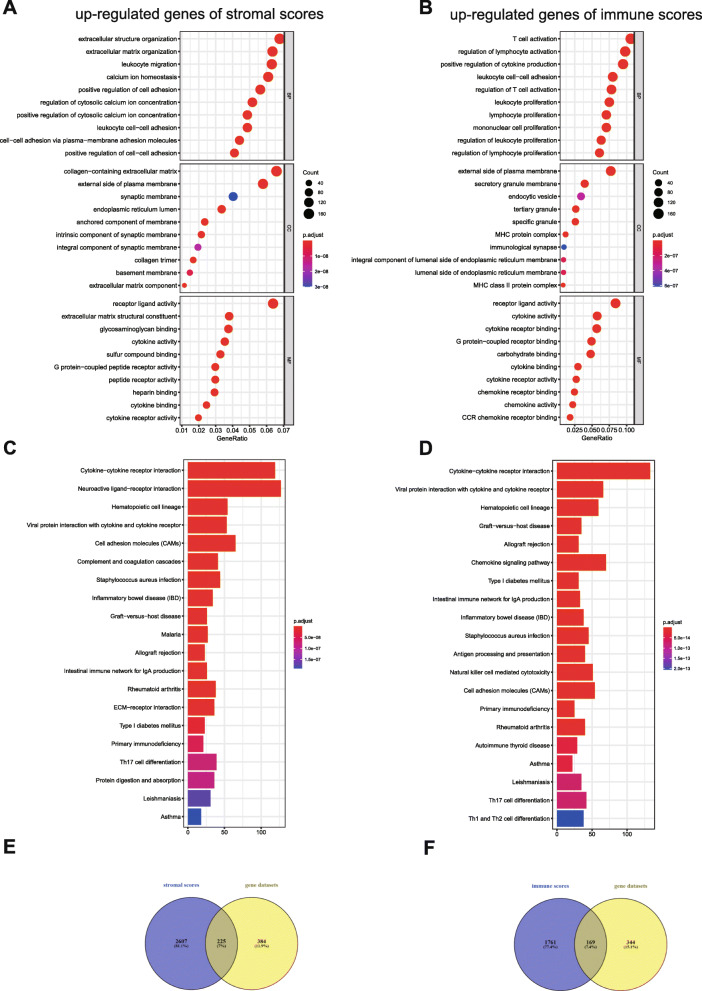
Differentially expressed genes (DEGs) for Gene Ontology (GO) enrichment and Kyto Encyclopedia of Genes and Genomes (KEGG) pathway analyses. (**A**) GO enrichment analysis of the upregulated genes in the high vs low stromal score groups; (**B**) GO enrichment analysis of the upregulated genes in the high vs low immune score groups; (**C**) KEGG pathway analysis of the upregulated genes in the high vs low stromal score groups; (**D**) KEGG pathway analysis of the upregulated genes in the high vs low immune score groups; (**E**) Venn diagrams showing the overlapping 1 genes; (**F**) Venn diagram showing the overlapping 2 genes

Next, in order to comprehend the function of DEGs, we selected the upregulated DEGs for GO enrichment and KEGG pathway analyses. The top GO terms identified for the upregulated DEGs from the high vs. low stromal score groups were extracellular structure organization, collagen-containing extracellular matrix, and receptor ligand activity (Fig. 4a). In addition, the top GO terms of the upregulated DEGs from the high vs. low immune score groups were T cell activation, external side of the plasma membrane, and receptor ligand activity (Fig. 4b). Moreover, the top KEGG pathways of the upregulated DEGs from the high vs. low stromal score and high vs. low immune score groups were neuroactive ligand-receptor interaction and cytokine-cytokine receptor interaction, respectively (Fig. 4c, d).

For deeply exploring the gene profiles of the top GO terms, we integrated the top 1 term of the BP, MF, or CC categories as gene datasets. Then, we screened the overlapping genes between upregulated DEGs and the gene datasets (Table S1). Venn diagrams showed that 225 genes overlapped between the upregulated genes from the high vs. low stromal score groups and the gene datasets (for convenience of description, we named these genes overlapping 1 genes), and 169 genes overlapped between the upregulated genes from the high vs. low immune score groups and the gene datasets (for convenience of description, we named these genes overlapping 2 genes) (Fig. 4e, f).

### The screened immune-related DEGs are associated with good prognosis in breast cancer

**Fig. 5:**
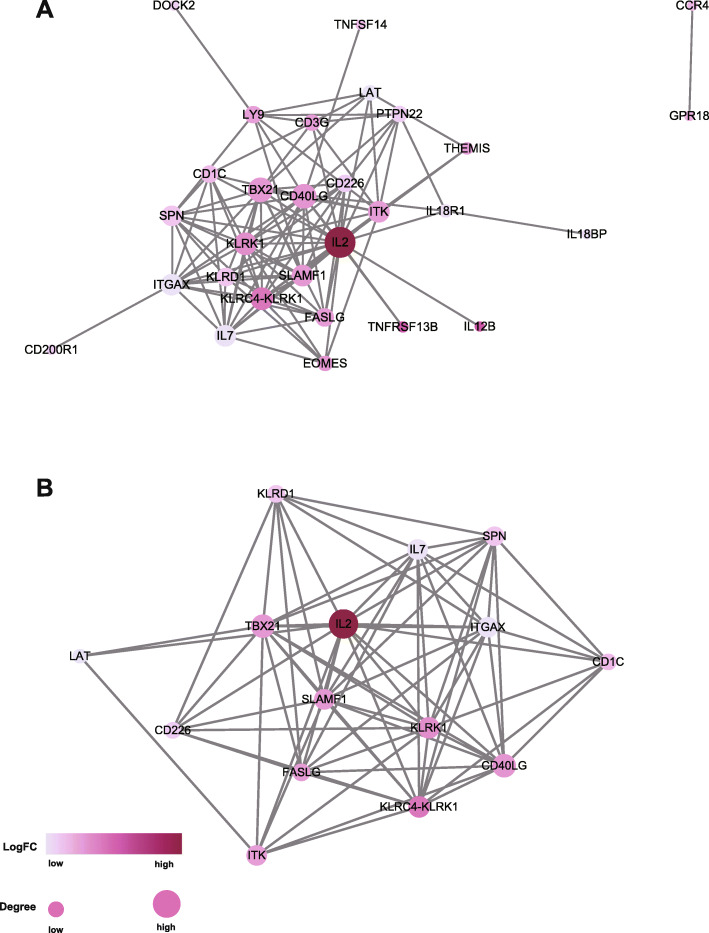
Protein-protein interaction (PPI) analysis of the overlapping immune-related genes. (**A**) PPI network of 31 screened genes, which included 29 nodes and 110 edges; (**B**) PPI network of 15 screened genes. The logFC value of the gene expression was used to reflect the color of each node, and the size of the node represents the number of proteins interacting with the designated protein

We performed a univariate survival analysis to reveal the relationship between the selected overlapping genes and prognosis in breast cancer patients from TCGA. Although the overlapping 1 genes did not show statistical significance in the OS analysis, the overlapping 2 genes exhibited highly significant differences in the prognosis of breast cancer, so we chose the overlapping 2 genes for further analysis. Using *p* value < 0.05, 54 genes related to good prognosis were included. Then, a time-dependent ROC curve (area under the curve (AUC) > 0.6) was employed to assess the prediction accuracy, and 31 genes with prognostic value in breast cancer remained. To better understand the interplay among the identified 31 genes, we performed PPI analysis, and Fig. 5a shows the overall PPI network, which included 29 nodes and 110 edges (Fig. 5a). Then, we selected the extremely critical module containing 15 nodes for further analysis (Fig. 5b).

**Fig. 6:**
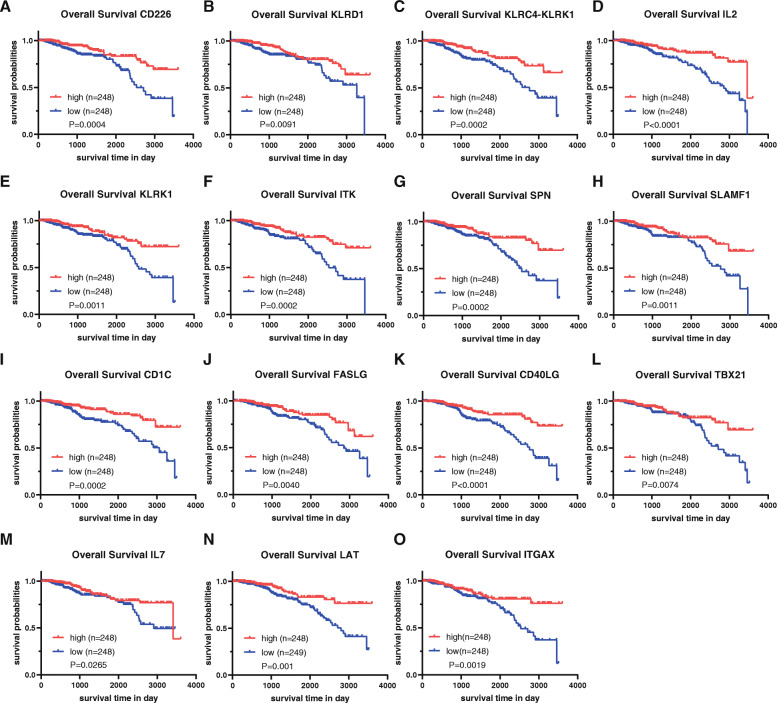
Kaplan-Meier survival curves for the 15 screened genes with good prognosis in breast cancer. The relationship between the expression of (**A**) CD226, (**B**) KLRD1, (**C**) KLRC4-KLRK1, (**D**) IL2, (**E**) KLRK1, (**F**) ITK, (**G**) SPN, (**H**) SLAMF1, (**I**) CD1C, (**J**) FASLG, (**K**) CD40LG, (**L**) TBX21, (**M**) IL7, (**N**) LAT, or (**O**) ITGAX and overall survival in breast cancer. *P* < 0.05 in the log-rank test

The 15 immune-related genes (CD226, KLRD1, KLRC4-KLRK1, IL2, KLRK1, ITK, SPN, SLAMF1, CD1C, FASLG, CD40LG, TBX21, IL7, LAT and ITGAX) in the TME that are associated with good prognosis are shown in Fig. 6a-o and Table 1.

**Table 1:** Genes with prognostic value

Gene symbol	HR	95% CI lower	95% CI upper	P value
CD226	0.6572	−0.6747	−0.1649	0.0004
KLRD1	0.6819	−0.6759	−0.0899	0.0091
KLRC4-KLRK1	0.3664	−1.8647	−0.1435	0.0002
IL2	0.2775	−2.1727	−0.3909	< 0.0001
KLRK1	0.5749	−1.0037	−0.1035	0.0011
ITK	0.9141	−0.1532	−0.0264	0.0002
SPN	0.9479	−0.0875	−0.0194	0.0002
SLAMF1	0.9197	−0.1436	−0.0239	0.0011
CD1C	0.9643	−0.0636	−0.0091	0.0002
FASLG	0.8986	−0.1904	−0.0234	0.0040
CD40LG	1.0678	0.0306	0.1006	< 0.0001
TBX21	0.8669	−0.2414	− 0.0442	0.0074
IL7	0.8524	−0.2990	−0.0204	0.0265
LAT	0.5610	−1.0788	−0.0772	0.0100
ITGAX	0.9127	−0.1514	−0.0313	0.0019

HR: Hazard ratio; CI: Confidence interval

**Fig. 7:**
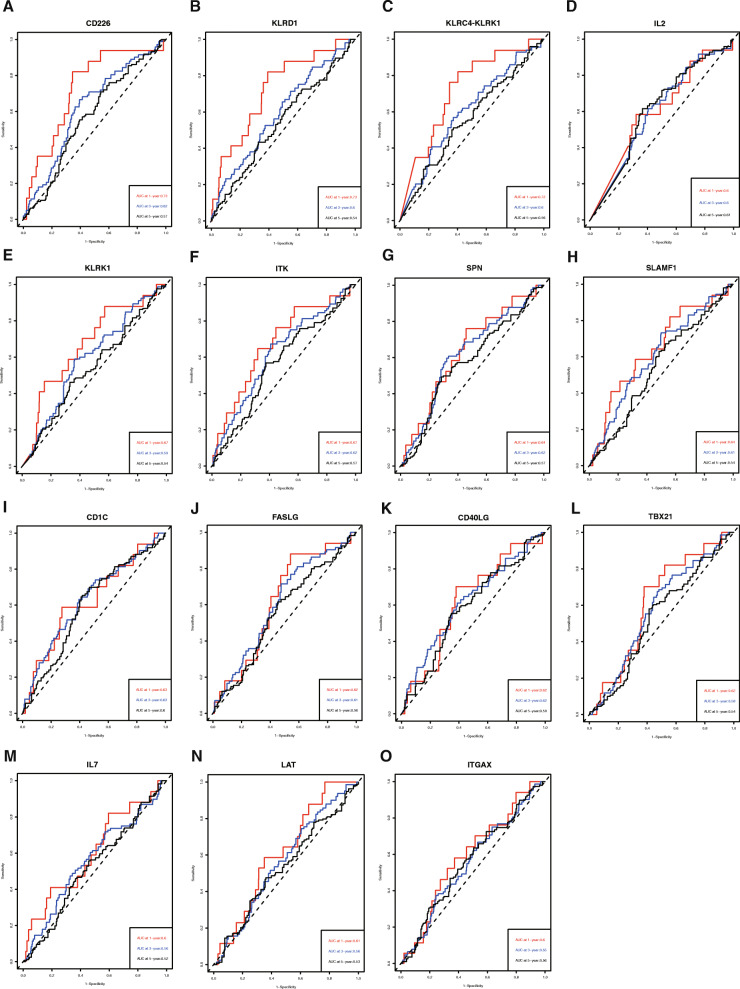
CD226, KLRD1 and KLRC4-KLRK1 had higher predictive accuracies than the other screened genes. Time-dependent ROC analyses were performed to compare the 15 screened gene signatures: (**A**) CD226, (**B**) KLRD1, (**C**) KLRC4-KLRK1, (**D**) IL2, (**E**) KLRK1, (**F**) ITK, (**G**) SPN, (**H**) SLAMF1, (**I**) CD1C, (**J**) FASLG, (**K**) CD40LG, (**L**) TBX21, (**M**) IL7, (**N**) LAT, and (**O**) ITGAX in predicting 1-year, 3-year and 5-year overall survival

Figure 7a-o displays the 15-gene signature of the time-dependent ROC curve, and their 95% confidence interval (CI) of AUC were shown in Table 2. The accuracies in predicting the 1-year, 3-year and 5-year OS of patients with CD226 (AUC 0.73, 0.62, and 0.57, respectively), KLRD1 (AUC 0.73, 0.6, and 0.54, respectively), and KLRC4-KLRK1 (AUC 0.72, 0.6, and 0.56, respectively) were higher than those with the other selected genes, especially for the 1-year prediction accuracy (Fig. 7 and Table 2). Therefore, we identified CD226, KLRD1, and KLRC4-KLRK1 for further functional evaluation.

**Table 2:** AUC (95% CI) of the 15 screened gene signatures in Time dependent ROC analyses

genes	AUC (95% CI)		
	1-year	3-year	5-year
CD226	0.73 (0.6136–0.8428)	0.62 (0.5533–0.6925)	0.57 (0.5007–0.6405)
KLRD1	0.73 (0.6079–0.8430)	0.60 (0.5235–0.6718)	0.54 (0.4685–0.6178)
KLRC4-KLRK1	0.72 (0.6063–0.8328)	0.60 (0.5295–0.6785)	0.56 (0.4868–0.6346)
IL2	0.60 (0.4710–0.7387)	0.60 (0.5310–0.6695)	0.61 (0.5418–0.6779)
KLRK1	0.67 (0.5400–0.7990)	0.59 (0.5220–0.6642)	0.54 (0.4681–0.6149)
ITK	0.67 (0.5445–0.8050)	0.62 (0.5503–0.6953)	0.57 (0.4975–0.6413)
SPN	0.64 (0.5094–0.7626)	0.62 (0.5468–0.6895)	0.57 (0.5014–0.6478)
SLAMF1	0.64 (0.5083–0.7764)	0.61 (0.5350–0.6804)	0.54 (0.4724–0.6145)
CD1C	0.63 (0.4879–0.7658)	0.63 (0.5577–0.7070)	0.60 (0.5286–0.6700)
FASLG	0.62 (0.5020–0.7360)	0.61 (0.5363–0.6765)	0.56 (0.4848–0.6297)
CD40LG	0.62 (0.4941–0.7477)	0.62 (0.5423–0.6935)	0.59 (0.5194–0.6602)
TBX21	0.62 (0.5021–0.7298)	0.58 (0.5084–0.6474)	0.54 (0.4651–0.6066)
IL7	0.60 (0.4624–0.7438)	0.56 (0.4839–0.6391)	0.52 (0.4531–0.5967)
LAT	0.61 (0.4995–0.7263)	0.56 (0.4917–0.6347)	0.53 (0.4583–0.6098)
ITGAX	0.59 (0.4649–0.7191)	0.55 (0.4806–0.6274)	0.56 (0.4878–0.6321)

### High expression of CD226 and KLRC4-KLRK1 results in a better prognosis in stage II, stage III or luminal B breast cancer

**Fig. 8:**
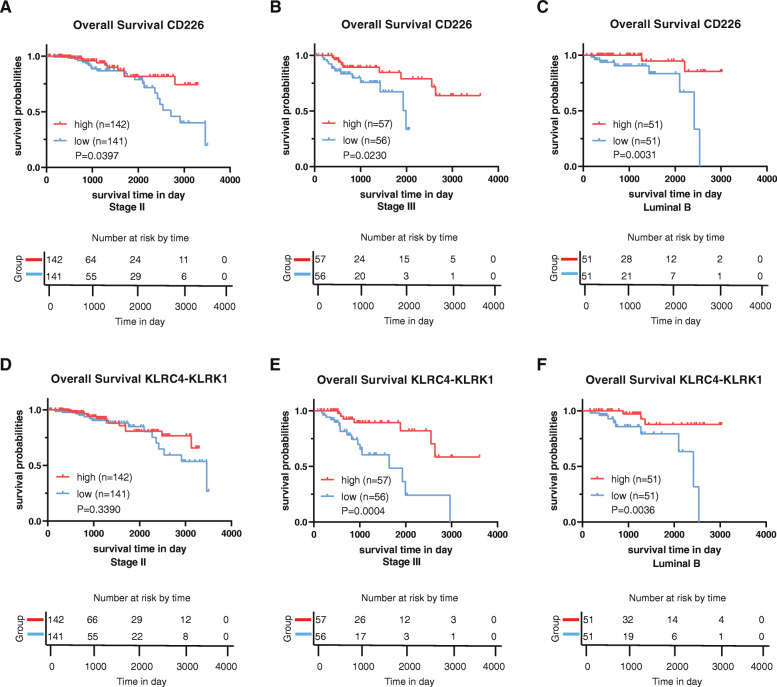
High expression of CD226 and KLRC4-KLRK1 results in a better prognosis in breast cancer. The survival probability differences between high and low CD226 expression in (**A**) stage II, (**B**) stage III, and (**C**) the luminal B subtype of breast cancer. The survival probability differences between high and low KLRC4-KLRK1 expression in (**D**) stage II, (**E**) stage III, and (**F**) the luminal B subtype of breast cancer. P < 0.05 in the log-rank test

To further explore the identified CD226, KLRD1, and KLRC4-KLRK1 genes with prognostic value, we analyzed the expression of these genes and their association with OS in different breast cancer stages or subtypes. Although the high expression of KLRD1 was not related to a better prognosis in any stage or subtype variation (data not shown), the expression of CD226 and KLRC4-KLRK1 displayed a strong correlation with OS for stages or subtypes. High CD226 expression was related to better prognosis in stage II and stage III breast cancer, as well as in luminal B breast cancer (Fig. 8a-c). Similarly, despite not being significant for stage II (Fig. 8d), high KLRC4-KLRK1 expression revealed preferable survival probabilities in stage III breast cancer and in the luminal B subtype (Fig. 8e, f). In other stages or subtypes of breast cancer, CD226 and KLRC4-KLRK1 expression was not significantly related to OS (Fig. S2).

### Validating that the prognostic genes identified from the TCGA database are equally significant in the GEO database

**Fig. 9:**
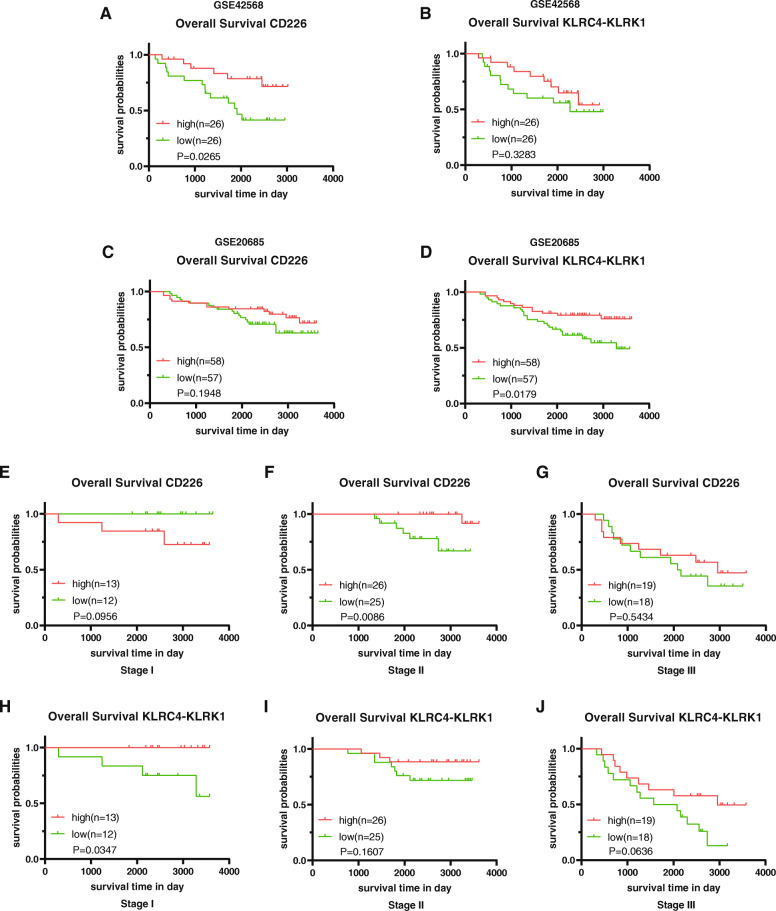
Validating the prognostic genes identified in the TCGA database are equally significant in GEO database. The relationship between (**A**) the expression of CD226 or (**B**) the expression of KLRC4-KLRK1 and breast cancer prognosis in the GSE42568 dataset; the relationship between (**C**) the expression of CD226 or (**D**) the expression of KLRC4-KLRK1 and breast cancer prognosis in the GSE20685 dataset; the relationship between the expression of CD226 and (**E**) stage I, (**F**) stage II, and (**G**) stage III breast cancer prognosis in the GSE20685 dataset; the relationship between the expression of KLRC4-KLRK1 and (**H**) stage I, (**I**) stage II, (**J**) stage III breast cancer prognosis in the GSE20685 dataset. P < 0.05 in the log-rank test

To validate whether the prognostic genes identified from the TCGA database are equally significant in the GEO database, two gene profiles from GEO (GSE20685 and GSE42568) were used as validation datasets. The gene expression data of 327 cases from GSE20685 and 104 cases from GSE42568 with clinical information were downloaded and evaluated for OS. Data from GSE42568 demonstrated that CD226 was related to good prognosis in breast cancer patients, while KLRC4-KLRK1 did not show any correlation with breast cancer OS (Fig. 9a, b). However, the data from GSE20685 showed the reversed results; instead of CD226, KLRC4-KLRK1 was related to good prognosis in breast cancer (Fig. 9c, d). Next, we employed the data from GSE20685 to better confirm the relationships between gene expression and OS in different cancer stages (Fig. 9e-j), and found CD226 was firmly associated with good prognosis in stage II breast cancer patients. Thus, our validation datasets partly proved that high CD226 and KLRC4-KLRK1 expression levels would predict satisfied overall survival of breast cancer, especially in specific stages.

## Discussion

The TME is crucial in tumor initiation, progression and drug resistance in breast cancer, and the infiltration of nontumor cells in the TME was calculated by the ESTIMATE algorithm. The ESTIMATE algorithm developed based on gene expression is valid and effective in various cancers, such as prostate cancer, breast cancer, and colon cancer [9, 10]. By utilizing the breast cancer cohorts in the TCGA database and ESTIMATE algorithm-derived scores, we first analyzed the relationship between stromal/immune scores and different stages or subtypes of breast cancer, found that stromal/immune scores were highly correlated with subtypes of breast cancer, especially in the basal-like subtype. In addition, the luminal B and HER2-enriched subtypes also showed relevance with immune scores. These findings demonstrated that TME variation correlated with subtypes of breast cancer and possessed immunological characteristics, which was consistent with the findings of a previous study [11]. However, the high immune-related scores did not predict a better prognosis in breast cancer subtypes. This is because prognosis is more influenced by various factors, such as age, race, never being pregnant or having a first child after age 30, type of surgical procedure, initial tumor size, clinical lymph mode status, and neoadjuvant chemotherapy [12, 13]. Moreover, when considering the tumor stroma, cancer-associated fibroblasts (CAFs) are the major constituent of tumor stroma, take part in induction of tumor progression in breast cancer. Mounting evidence indicates that CAFs are heterogeneous, like tumor cells, among different subtypes or in the same subtypes of breast cancer which patients have different length of survival [14, 15]. Currently, the potential role of CAFs as a predictive biomarker of tumor prognosis is still debated.

Second, we analyzed the relationship between the TME and gene mutations. Although our data demonstrated that CDH1, TP53 and PTEN mutations were closely associated with the TME, BRCA1 and BRCA2 mutations did not show significant differences in TME variation. BRCA1 and BRCA2 mutant genes predispose individuals to an elevated risk of breast cancer, and those with a family history of cancer are recommended to undergo gene detection based on the National Comprehensive Cancer Network (NCCN) guidelines [16]. Thus, BRCA1 and BRCA2 mutations are not highly frequent in sporadic cases, the design of upcoming trials stratifying patients by BRCA status to avoid potential bias are needed. CDH1, TP53 and PTEN are tumor suppressor genes, their alteration presents poor survival and worse prognosis in breast cancer [17–19]. Our study findings coincide with those of a previous exploration that after the exclusion of BRCA1 or BRCA2, the TME correlates with TP53, CDH1, and PTEN mutations, their mutations even revealed a highly or moderately increased risk of breast cancer [20].

Third, when further exploring the DEGs in the TME, many previous studies have ignored genes with low expression but exhibit high significance in antitumor activity. Thus, to eliminate the factor of tumor cells downregulating the expression of antitumor genes during tumor progression, we focused on the genes of the top GO terms of the upregulated DEGs based on functional enrichment.

Fourth, through step-by-step screening via OS analysis, time-dependent ROC analysis, and PPI network analysis, we revealed that the top GO term genes of the upregulated DEGs from the high vs low immune score groups exhibited better prognosis in breast cancer, which could be explained by the fact that the immune system plays an important role in cancer development and therefore potentially offers novel targeted therapies in antitumor treatment [21]. Ultimately, we found 2 important TME genes with good prognosis (CD226 and KLRC4-KLRK1).

CD226, also known as DNAM-1, is an activating receptor expressed on various immune cells, such as CD4+ and CD8+ T lymphocytes, regulatory T cells (Tregs), monocytes, macrophages, and NK cells [22, 23]. CD226 serves as a costimulator, enhances T cell or NK cell activation [22], and exhibits significance in innate/adaptive immune regulatory networks. When combined with its ligand CD115 or CD112 upregulated in tumor cells [24], CD226 facilitates the cytotoxicity of NK cells [25]. Moreover, in Treg-mediated tumor immune escape, Tregs express relatively high levels of TIGIT and low levels of CD226 compared with effector T cells (Teffs), resulting in a high ratio of TIGIT/CD226 expression and accelerating tumor development. In contrast, augmenting CD226 expression and reversing the TIGIT/CD226 ratio would predict a good clinical outcome [26]. However, reduction in CD226 expression decreases the immune regulatory capacity, and CD226-deficient CTL or NK cells exhibit markedly less cytotoxic activity against DNAM-1 ligand-expressing tumors [27]. Accumulating evidence has shown that CD226 plays a pivotal role in tumor recognition and cancer immune surveillance [28], even promoting antitumor immune responses mediated by NK and T cells.

Killer cell lectin-like receptor subfamily C4 - killer cell lectin-like receptor subfamily K1 (KLRC4-KLRK1) belongs to killer cell lectin-like receptor family [29] and represents naturally occurring read-through transcription between neighboring KLRC4 and KLRK1. KLRC4 lacks a significant portion of the KLRC4 coding sequence but encodes the KLRK1 protein. Once tumorigenesis occurs, the amount of KLRK1 ligand increases immediately. KLRK1 (or NKG2D) is also an activating receptor expressed by NK cells and T cell subsets, can augment the cytotoxicity of NK cells/T cells or synergize with immune checkpoint inhibitors to eliminate tumor cells [30]. However, tumor cells evoke a range of mechanisms to evade KLRK1 surveillance system detection and impair the clinical benefits of immunotherapy in various cancers [31, 32]. The downregulation of KLRK1 hampered NK cell cytotoxicity [33]. Conversely, blocking the shedding of ligands by tumors or the release of KLRK1 ligand-bearing exosomes might restore the expression of KLRK1 receptors on NK cells or T cells and improve their activity [34].

It was reported that the KLRK1 axis is becoming an emerging target in cancer immunotherapy [35, 36], and the overexpression of CD226 or KLRK1 on NK cells resulted in efficient anti-sarcoma activity [37]. These studies were in accordance with our exploration to provide a molecular basis for the development of CD226- and KLRC4-KLRK1-targeted antitumor immune therapeutics. Considering that the samples from GEO database were much fewer than those from TCGA database, further exploration should include in more breast cancer patients for verification.

Tumorigenesis is initiated by 3 steps: cancer cell elimination by various immune cells, such as NK cells and CD8+ T cells, then immune pressure leading to the selection of tumor cell variants and finally, immune escape by inhibiting effector cells or inducing tolerogenic cells [38]. Escaping antitumor immunity is a hallmark for the progression of breast cancer. In the TME, tumor cells interact with various types of immune cells by activating immune checkpoint pathways [39, 40]. Immune checkpoints (CTLA-4, PD-1/PDL1, LAG3, TIM3 and TIGIT) are orchestrated by a series of costimulatory and inhibitory signaling molecules and then modulate effector T lymphocyte (Teff) activity. Recent advancements in antibodies against immune checkpoints have highlighted the benefits of immune checkpoint inhibitors in both animal studies and clinical trials [41].

In this study, we also detected immune checkpoint genes, including CTLA-4, PD-1, LAG3 and TIM3, and found that their high expression was not associated with good prognosis in breast cancer (data not shown). This finding is probably related to the level of checkpoint gene expression and immune state of the TME [8]. To achieve unbiased results, the identification of immune checkpoint gene expression level is critical. The investigation into the relationship between immune checkpoint gene expression and the TME immune state (such as tumor-infiltrating lymphocytes and the T cell receptor repertoire) would provide key insights into checkpoint blockade therapy.

At present, there are multiple antitumor approaches: inverting tumor immunosuppression (for example, employing immune checkpoint inhibitors and the direct induction of the Teff immune response), using immune-based therapies targeting specific immune cell types (including improving cytotoxic efficacy, and promoting immune surveillance through NK cells or Teffs), reducing the number of immunosuppressive myeloid cells, inhibiting Tregs, and altering the function of myeloid cells [7].

Despite combining multidisciplinary treatment strategies, breast cancer patients still have a comparably high mortality. An improved understanding of the immune-related genetic profile of TME in breast cancer and the identification of new immunological targets are critical for improving clinical outcomes. CD226, KLRC4-KLRK1 and subsequent new targets seem to be promising avenues for promoting antitumor targeted therapy in breast cancer.

## Conclusions

The exploration of the TME and immunological treatment in breast cancer have become increasingly important in recent years. In our study, although the TME did not correlate with the stages of breast cancer, we verified that it was highly associated with the subtypes of breast cancer and gene mutations (CDH1, TP53 and PTEN) and possessed immunological characteristics. The combined analysis of OS, time-dependent ROC, and the PPI network revealed that the genes of the top 3 GO terms of the upregulated DEGs from the high vs. low immune score groups were associated with better prognosis in breast cancer, and 15 of them were related to good prognosis in breast cancer, especially CD226 and KLRC4-KLRK1. High CD226 and KLRC4-KLRK1 expression levels were identified, and their correlation with better OS in specific stages or subtypes of breast cancer was validated.

## Supplementary Information

**Additional file 1 Fig. S1**. The high and low stromal/immune score groups did not show any survival differences in the four breast cancer subtypes. The high and low stromal score groups’ survival probability differences in the (**A**) luminal A, (**B**) luminal B, (**C**) HER2-enriched, (**D**) basal-like subtypes. The high and low immune score groups’ survival probability differences in the (**E**) luminal A, (**F**) luminal B, (**G**) HER2-enriched, and (**H**) basal-like subtypes. *P* < 0.05 in the log-rank test.

**Additional file 2 Fig. S2**. The high and low expression of CD226 and KLRC4-KLRK1 did not show any survival differences in stage I, the luminal A subtype, the HER2-enriched subtype or the basal-like subtype of breast cancer. The survival probability differences of high and low (**A**) CD226 and (**B**) KLRC4-KLRK1 expression in stage I breast cancer; the survival probability differences of high and low CD226 expression in the (**C**) luminal A, (**D**) luminal B, and (**E**) HER2-enriched subtypes of breast cancer; the survival probability differences of high and low KLRC4-KLRK1 expression in the (**F**) luminal A, (**G**) luminal B, and (**H**) HER2-enriched subtypes of breast cancer. P < 0.05 in the log-rank test.

**Additional file 3 Table S1**. The overlapping genes between the upregulated DEGs from the high vs low immune score groups and the gene datasets.

## Abbreviations

DEGs: differentially expressed genes
ESTIMATE: Estimation of stromal and immune cells in malignant tumors using expression data
GO: Gene Ontology
TCGA: The Cancer Genome Atlas
TPM: transcripts per kilobase million
KEGG: Kyoto Encyclopedia of Genes and Genomes

## References

[1] Torre LA, Bray F, Siegel RL, Ferlay J, Lortet-Tieulent J, Jemal A (2015). Global cancer statistics, 2012. CA Cancer J Clin.

[2] Lu S-N, Burkhamer J, Kriebel D, Clapp R. The increasing toll of adolescent cancer incidence in the US. PLoS One. 2017:12(2). 10.1371/journal.pone.0172986 PMC5325567 28235028

[3] Siegel RL, Miller KD, Jemal A (2019). Cancer statistics, 2019. CA Cancer J Clin.

[4] Olver IN (2016). New initiatives in the treatment of breast cancer. Med J Aust.

[5] Bussard KM, Mutkus L, Stumpf K, Gomez-Manzano C, Marini FC. Tumor-associated stromal cells as key contributors to the tumor microenvironment. Breast Cancer Res. 2016:18(1). 10.1186/s13058-016-0740-2 PMC4982339 27515302

[6] Hui L, Chen Y (2015). Tumor microenvironment: sanctuary of the devil. Cancer Lett.

[7] Soysal SD, Tzankov A, Muenst SE (2015). Role of the tumor microenvironment in breast Cancer. Pathobiology.

[8] Gibney GT, Weiner LM, Atkins MB (2016). Predictive biomarkers for checkpoint inhibitor-based immunotherapy. The Lancet Oncology.

[9] Yoshihara K, Shahmoradgoli M, Martinez E, Vegesna R, Kim H, Torres-Garcia W, Trevino V, Shen H, Laird PW, Levine DA (2013). Inferring tumour purity and stromal and immune cell admixture from expression data. Nat Commun.

[10] Donovan MJ, Fernandez G, Scott R, Khan FM, Zeineh J, Koll G, Gladoun N, Charytonowicz E, Tewari A, Cordon-Cardo C (2018). Development and validation of a novel automated Gleason grade and molecular profile that define a highly predictive prostate cancer progression algorithm-based test. Prostate Cancer Prostatic Dis.

[11] Xie P, Ma Y, Yu S, An R, He J, Zhang H (2019). Development of an immune-related prognostic signature in breast Cancer. Front Genet.

[12] Tejera Hernández AA, Vega Benítez VM, Rocca Cardenas JC, Gutiérrez Giner MI, Díaz Chico JC, Hernández Hernández JR (2019). Factors predicting local relapse and survival in patients treated with surgery for breast cancer. Asian Journal of Surgery.

[13] Campbell JB (2002). Breast cancer-race, ethnicity, and survival: a literature review. Breast Cancer Res Treat.

[14] Mao Y, Keller ET, Garfield DH, Shen K, Wang J (2013). Stromal cells in tumor microenvironment and breast cancer. Cancer Metastasis Rev.

[15] Finak G, Bertos N, Pepin F, Sadekova S, Souleimanova M, Zhao H, Chen H, Omeroglu G, Meterissian S, Omeroglu A (2008). Stromal gene expression predicts clinical outcome in breast cancer. Nat Med.

[16] Daly MB, Pilarski R, Berry M, Buys SS, Farmer M, Friedman S, Garber JE, Kauff ND, Khan S, Klein C (2017). NCCN guidelines insights: genetic/familial high-risk assessment: breast and ovarian, version 2.2017. J Natl Compr Cancer Netw.

[17] Corso G, Intra M, Trentin C, Veronesi P, Galimberti V (2016). CDH1 germline mutations and hereditary lobular breast cancer. Familial Cancer.

[18] Olivier M (2006). The clinical value of somatic TP53 gene mutations in 1,794 patients with breast Cancer. Clin Cancer Res.

[19] Ngeow J, Sesock K, Eng C (2015). Breast cancer risk and clinical implications for germline PTEN mutation carriers. Breast Cancer Res Treat.

[20] Couch FJ, Shimelis H, Hu C, Hart SN, Polley EC, Na J, Hallberg E, Moore R, Thomas A, Lilyquist J (2017). Associations between Cancer predisposition testing panel genes and breast Cancer. JAMA Oncol.

[21] Gajewski TF, Corrales L, Williams J, Horton B, Sivan A, Spranger S: Cancer Immunotherapy Targets Based on Understanding the T Cell-Inflamed Versus Non-T Cell-Inflamed Tumor Microenvironment. In: *Tumor Immune Microenvironment in Cancer Progression and Cancer Therapy.* edn.; 2017: 19–31. 10.1007/978-3-319-67577-0_2 PMC6693322 29275462

[22] Lenac Rovis T, Kucan Brlic P, Kaynan N, Juranic Lisnic V, Brizic I, Jordan S, Tomic A, Kvestak D, Babic M, Tsukerman P (2016). Inflammatory monocytes and NK cells play a crucial role in DNAM-1–dependent control of cytomegalovirus infection. J Exp Med.

[23] Gross CC (2017). Meyer zu Hörste G, Schulte-Mecklenbeck a, Klotz L, Meuth SG, Wiendl H: reply to Liu et al.: haplotype matters: CD226 polymorphism as a potential trigger for impaired immune regulation in multiple sclerosis. Proc Natl Acad Sci.

[24] Wang H, Qi J, Zhang S, Li Y, Tan S, Gao GF (2019). Binding mode of the side-by-side two-IgV molecule CD226/DNAM-1 to its ligand CD155/Necl-5. Proc Natl Acad Sci.

[25] Li Y, Yang F, Zhu J, Sang L, Han X, Wang D, Shan F, Li S, Sun X, Lu C (2015). CD226 as a genetic adjuvant to enhance immune efficacy induced by Ag85A DNA vaccination. Int Immunopharmacol.

[26] Fourcade J, Sun Z, Chauvin J-M, Ka M, Davar D, Pagliano O, Wang H, Saada S, Menna C, Amin R, et al. CD226 opposes TIGIT to disrupt Tregs in melanoma. JCI Insight. 2018:3(14). 10.1172/jci.insight.121157 PMC6124410 30046006

[27] Shibuya A, Shibuya K, Kikutani H, Yasui T, Honda S-I, Tahara-Hanaoka S, Shibata K, Yamashita Y, Kai H (2008). Iguchi-Manaka a: accelerated tumor growth in mice deficient in DNAM-1 receptor. J Exp Med.

[28] Guillamón CF, Martínez-Sánchez MV, Gimeno L, Mrowiec A, Martínez-García J, Server-Pastor G, Martínez-Escribano J, Torroba A, Ferri B, Abellán D (2018). NK cell education in tumor immune surveillance: DNAM-1/KIR receptor ratios as predictive biomarkers for solid tumor outcome. Cancer Immunology Research.

[29] Salmaninejad A, Zamani MR, Shabgah AG, Hosseini S, Mollaei F, Hosseini N, Sahebkar A (2018). Behçet’s disease: An immunogenetic perspective. J Cell Physiol.

[30] von Linsingen R, Pinho de Franca P, de Carvalho NS, MDG B. MICA and KLRK1 genes and their impact in cervical intraepithelial neoplasia development in the southern Brazilian population. Hum Immunol. 2020. 10.1016/j.humimm.2020.02.007 32107037

[31] Zuo J, Willcox CR, Mohammed F, Davey M, Hunter S, Khan K, Antoun A, Katakia P, Croudace J, Inman C, et al. A disease-linked ULBP6 polymorphism inhibits NKG2D-mediated target cell killing by enhancing the stability of NKG2D ligand binding. Sci Signal. 2017:10(481). 10.1126/scisignal.aai8904 28559451

[32] López-Soto A, Huergo-Zapico L, Acebes-Huerta A, Villa-Alvarez M, Gonzalez S (2015). NKG2D signaling in cancer immunosurveillance. Int J Cancer.

[33] Ashiru O, Boutet P, Fernandez-Messina L, Aguera-Gonzalez S, Skepper JN, Vales-Gomez M, Reyburn HT (2010). Natural killer cell cytotoxicity is suppressed by exposure to the human NKG2D ligand MICA*008 that is shed by tumor cells in exosomes. Cancer Res.

[34] Lanier LL (2015). NKG2D receptor and its ligands in host defense. Cancer Immunol Res.

[35] Hofer E, Sobanov Y, Brostjan C, Lehrach H, Duchler M (2001). The centromeric part of the human natural killer (NK) receptor complex: lectin-like receptor genes expressed in NK, dendritic and endothelial cells. Immunol Rev.

[36] Schmiedel D, Mandelboim O. NKG2D ligands–critical targets for Cancer immune escape and therapy. Front Immunol. 2018;9. 10.3389/fimmu.2018.02040 PMC6141707 30254634

[37] Sayitoglu EC, Georgoudaki A-M, Chrobok M, Ozkazanc D, Josey BJ, Arif M, Kusser K, Hartman M, Chinn TM, Potens R, et al. Boosting natural killer cell-mediated targeting of sarcoma through DNAM-1 and NKG2D. Front Immunol. 2020;11. 10.3389/fimmu.2020.00040 PMC7001093 32082316

[38] Galon J, Bruni D (2020). Tumor immunology and tumor evolution: intertwined histories. Immunity.

[39] Tan S, Xia L, Yi P, Han Y, Tang L, Pan Q, Tian Y, Rao S, Oyang L, Liang J (2020). Exosomal miRNAs in tumor microenvironment. J Exp Clin Cancer Res.

[40] Solinas C, Gombos A, Latifyan S, Piccart-Gebhart M, Kok M, Buisseret L. Targeting immune checkpoints in breast cancer: an update of early results. ESMO Open. 2017:2(5). 10.1136/esmoopen-2017-000255 PMC5687552 29177095

[41] Shi T, Ma Y, Yu L, Jiang J, Shen S, Hou Y, Wang T. Cancer Immunotherapy: A Focus on the Regulation of Immune Checkpoints. Int J Mol Sci. 2018:19(5). 10.3390/ijms19051389 PMC5983802 29735917

