# Supplementary figures and images for: Novel immune-related genes in the tumor microenvironment with prognostic value in breast cancer
Source: BMC Cancer. 2021 Feb 6;21:126. doi: 10.1186/s12885-021-07837-1 (PMC7866632; doi:10.1186/s12885-021-07837-1)

Group: — high stromal score — low stromal score

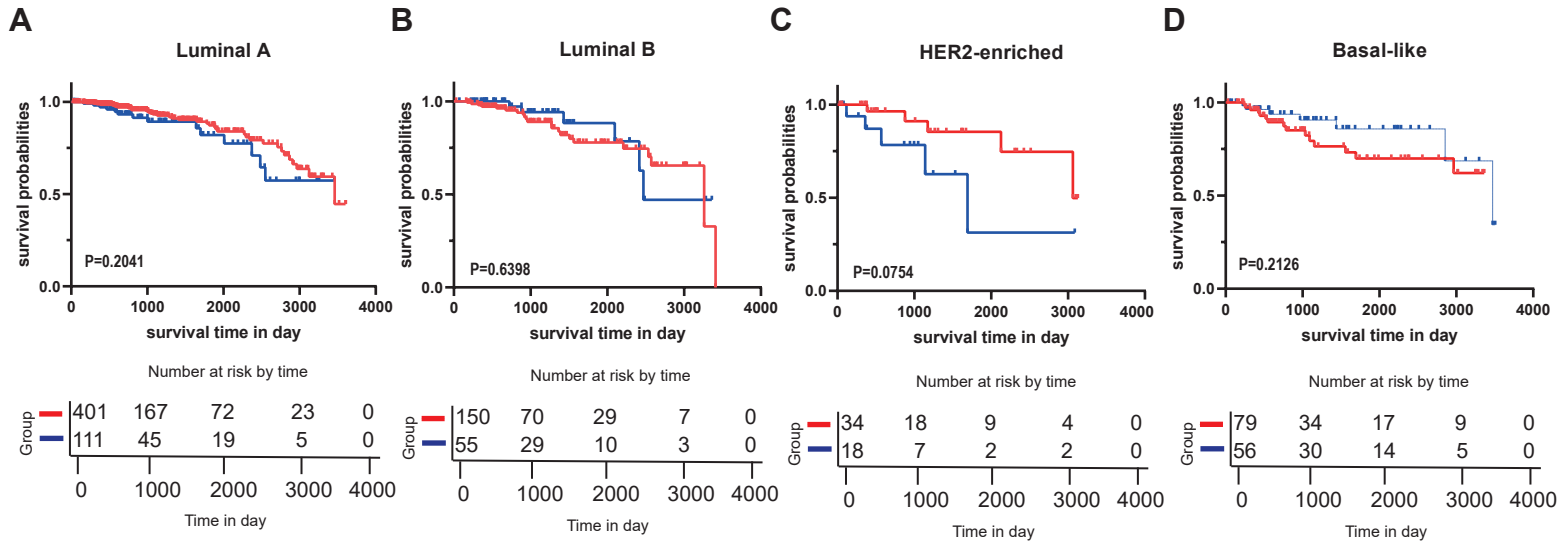

Group: — high immune score — low immune score

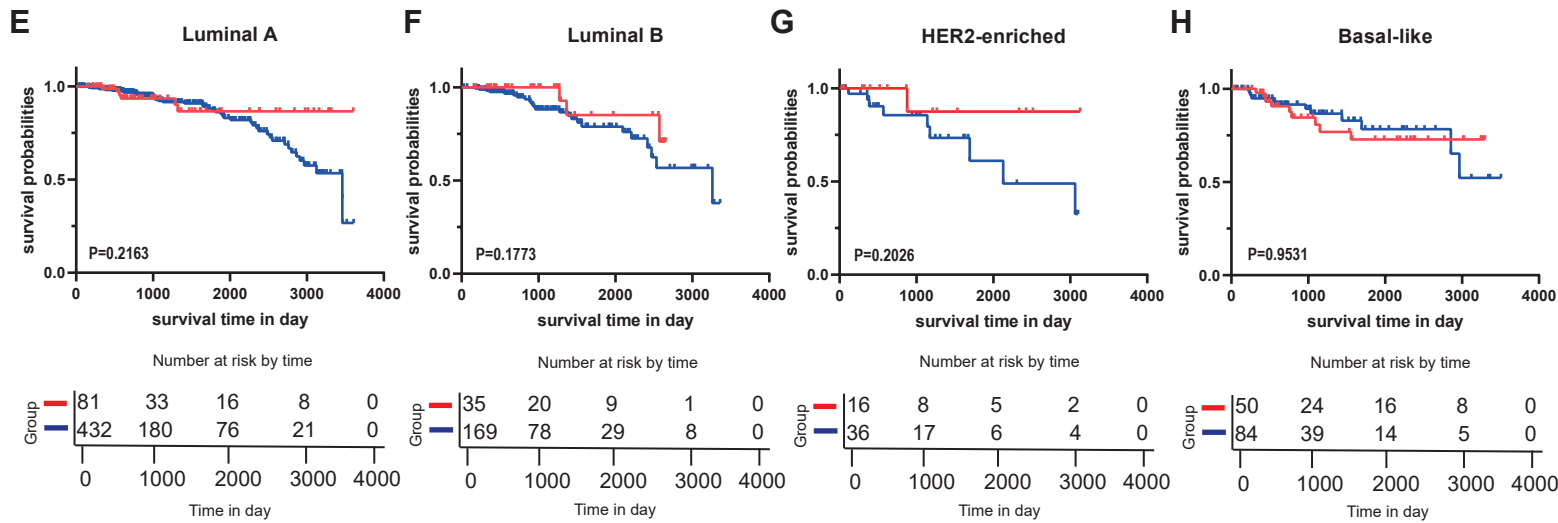

Supplement: Supplementary file 1 — Additional file 1 Fig. S1. The high and low stromal/immune score groups did not show any survival differences in the four breast cancer subtypes. The high and low stromal score groups’ survival probability differences in the (A) luminal A, (B) luminal B, (C) HER2-enriched, (D) basal-like subtypes. The high and low immune score groups’ survival probability differences in the (E) luminal A, (F) luminal B, (G) HER2-enriched, and (H) basal-like subtypes. P < 0.05 in the log-rank test. [file 12885_2021_7837_MOESM1_ESM.pdf]

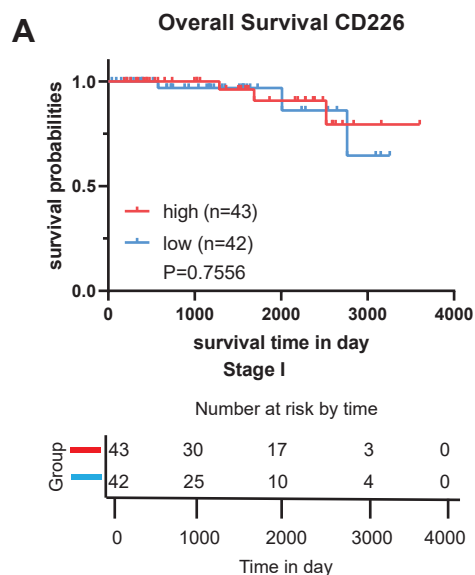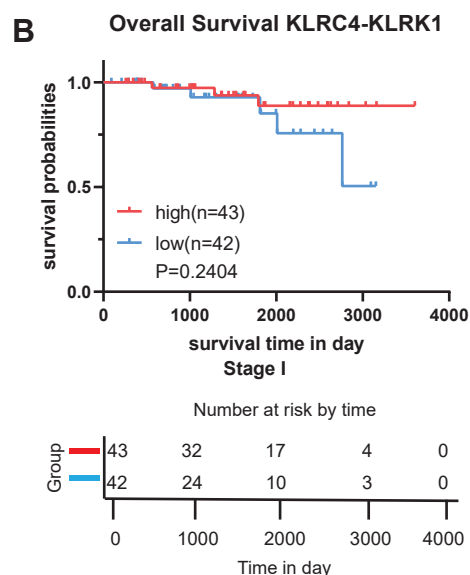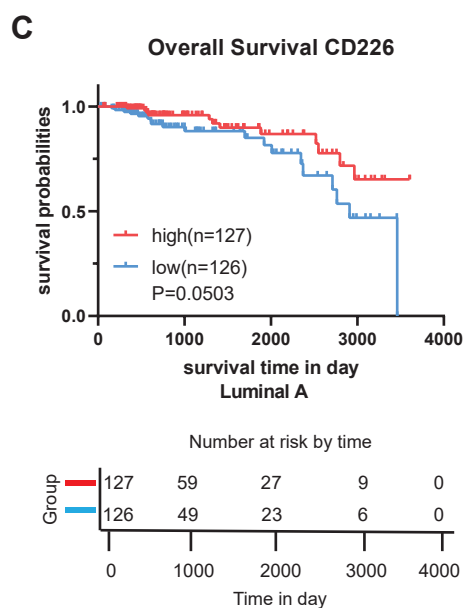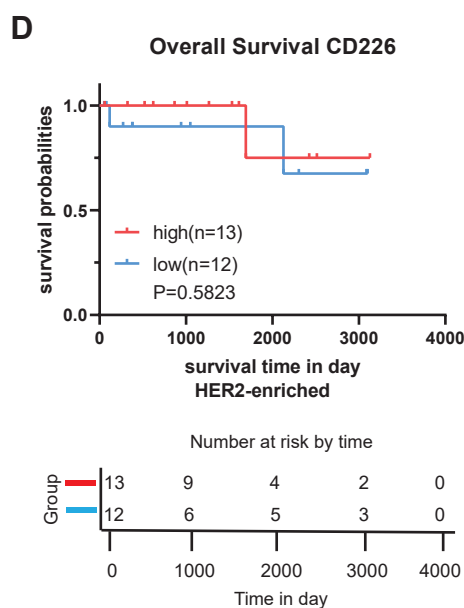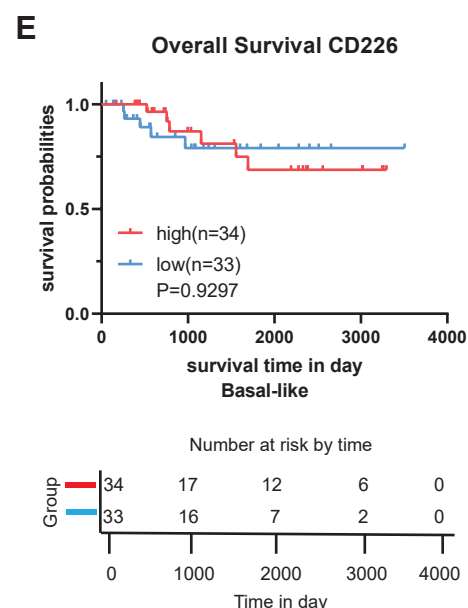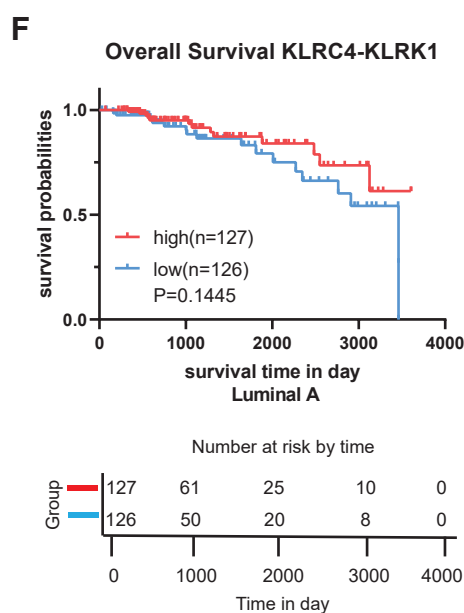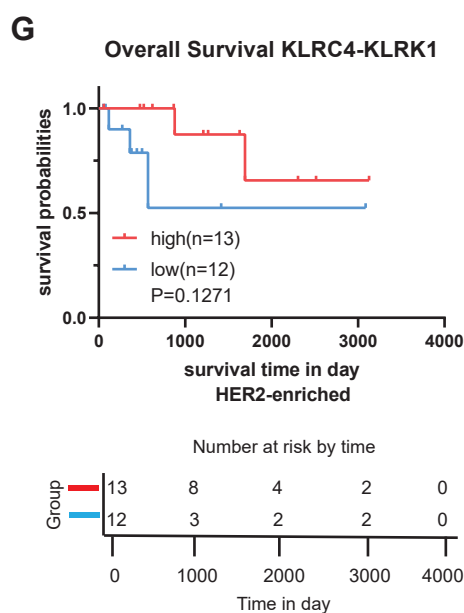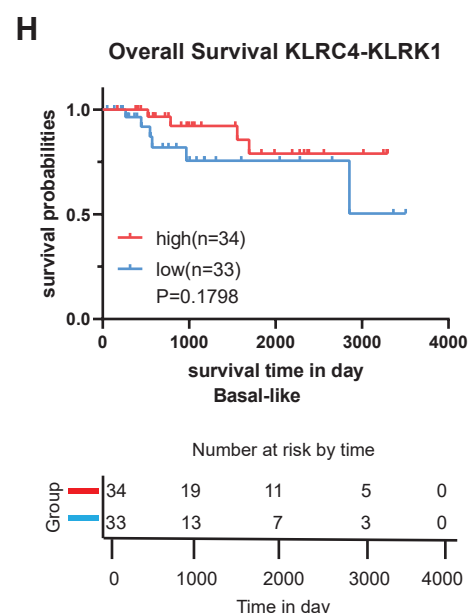

Supplement: Supplementary file 2 — Additional file 2 Fig. S2. The high and low expression of CD226 and KLRC4-KLRK1 did not show any survival differences in stage I, the luminal A subtype, the HER2-enriched subtype or the basal-like subtype of breast cancer. The survival probability differences of high and low (A) CD226 and (B) KLRC4-KLRK1 expression in stage I breast cancer; the survival probability differences of high and low CD226 expression in the (C) luminal A, (D) luminal B, and (E) HER2-enriched subtypes of breast cancer; the survival probability differences of high and low KLRC4-KLRK1 expression in the (F) luminal A, (G) luminal B, and (H) HER2-enriched subtypes of breast cancer. P < 0.05 in the log-rank test. [file 12885_2021_7837_MOESM2_ESM.pdf]
