# Supplementary material for: Novel immune-related genes in the tumor microenvironment with prognostic value in breast cancer
Source: BMC Cancer. 2021 Feb 6;21:126. doi: 10.1186/s12885-021-07837-1 (PMC7866632; doi:10.1186/s12885-021-07837-1)
Supplement: Supplementary file 3 — Additional file 3 Table S1. The overlapping genes between the upregulated DEGs from the high vs low immune score groups and the gene datasets. [file 12885_2021_7837_MOESM3_ESM.docx]

Table S1. The overlapping genes between the upregulated DEGs from the high vs low immune score groups and the gene datasets

| ADH7 | CD69 | HLA-DRB5 | NKX2-3 |
| --- | --- | --- | --- |
| AIRE | CD7 | HLA-E | NLRC3 |
| APBB1IP | CD74 | ICAM1 | P2RX7 |
| B2M | CD79A | ICOS | P2RY12 |
| BCL11B | CD79B | IFNB1 | PDCD1 |
| BTN3A1 | CD80 | IFNE | PIK3CD |
| CA4 | CD83 | IFNG | PIK3CG |
| CARD11 | CD86 | IGLL1 | PKHD1 |
| CCL11 | CD8A | IGLL5 | PLA2G2D |
| CCL19 | CD8B | IL12B | PLET1 |
| CCL21 | CLEC4D | IL12RB1 | PTGER4 |
| CCL24 | CLEC4E | IL12RB2 | PTPN22 |
| CCL5 | CLEC7A | IL13 | PTPRC |
| CCR1 | CRTAM | IL15 | RSAD2 |
| CCR4 | CTLA4 | IL17A | SELL |
| CCR5 | CXCL10 | IL18BP | SELP |
| CCR6 | CXCL13 | IL18R1 | SLA2 |
| CCR7 | CXCL9 | IL2 | SLAMF1 |
| CCR9 | CXCR3 | IL2RA | SLAMF6 |
| CD14 | CXCR5 | IL2RB | SPN |
| CD163 | DKK1 | IL2RG | TBX21 |
| CD19 | DNAI2 | IL31RA | TCF7 |
| CD1C | DOCK2 | IL36RN | THEMIS |
| CD1D | DOCK8 | IL6 | TLR8 |
| CD2 | EBI3 | IL7 | TMC1 |
| CD200R1 | EOMES | IL7R | TNF |
| CD200R1L | EPHB6 | IRF1 | TNFRSF13B |
| CD209 | FASLG | IRF4 | TNFRSF13C |
| CD226 | FCER1G | ITGAL | TNFRSF4 |
| CD244 | FCER2 | ITGAX | TNFRSF9 |
| CD27 | FCGR3A | ITK | TNFSF14 |
| CD274 | FCN1 | KLRC4-KLRK1 | TNFSF18 |
| CD28 | FCRL6 | KLRD1 | TNFSF8 |
| CD33 | FGA | KLRK1 | TREML2 |
| CD3D | FGG | LAG3 | TRPM8 |
| CD3E | FOLR2 | LAT | TXK |
| CD3G | FOXP3 | LCK | VAV1 |
| CD4 | FUT7 | LCP1 | VCAM1 |
| CD40 | GP1BA | LEP | WAS |
| CD40LG | GPR18 | LILRB1 | WNT1 |
| CD48 | GPR183 | LY9 | WNT10B |
| CD5 | HLA-DRB1 | MS4A1 | WNT7A |
|  |  |  | ZAP70 |
